# Supplementary material for: Transcutaneous Auricular Vagus Nerve Stimulation Facilitates Cortical Arousal and Alertness
Source: Int J Environ Res Public Health. 2023 Jan 12;20(2):1402. doi: 10.3390/ijerph20021402 (PMC9859411; doi:10.3390/ijerph20021402)
Supplement: Supplementary file 1 [file ijerph-20-01402-s001.zip › ijerph-2124347-supplementary.pdf]

Supplementary Materials:

Table S1. Descriptive statistics of participants' demographic information and emotional inventories and the statistical comparison between the taVNS group and the control group.

	Mean (SD)		t	p
	taVNS	Control		
Age (year)	23.170(2.879)	22.770(3.319)	0.501	0.618
Years of Education (year)	16.280(2.068)	15.800(1.883)	0.925	0.359
taVNS Stimulus Intensity (μA)	371.380(172.062)	389.000(227.131)	-0.335	0.739
BDI-Ⅱ	8.620 (9.959)	7.870 (8.059)	0.320	0.750
T-AI	40.590 (11.990)	39.23 (8.232)	0.504	0.617

taVNS, transcutaneous auricular vagus nerve stimulation; BDI-Ⅱ: Chinese version of the Beck Depression Inventory-Ⅱ; T-AI, Trait-Anxiety Inventory subscale from the State-Trait Anxiety Inventory. SD, standard deviation.

Table S2. Descriptive statistics and the statistical comparison between the taVNS group and the control group for the characteristics of the microstates under the close-eye resting state.

			Mean (SD)		t	p
			taVNS	Control		
Post−Pre Differences for Microstate A	Occurrence Rate (Hz)		-0.008 (0.322)	-0.134 (0.490)	1.164	0.249
	Mean Duration (ms)		0.691 (6.621)	0.465 (6.085)	0.137	0.892
	Time Coverage (%)		0.296 (2.723)	-0.737 (3.859)	1.184	0.241
	Transition Probabilities (*10^3)	To B	-1.189 (10.637)	-0.019 (10.817)	-0.419	0.677
		To C	3.657 (13.547)	0.012 (14.396)	1.001	0.321
		To D	0.369 (11.687)	-3.534 (12.653)	1.230	0.224
Post−Pre Differences for Microstate B	Occurrence Rate (Hz)		-0.068 (0.405)	0.040 (0.296)	-1.172	0.246
	Mean Duration (ms)		1.868 (6.689)	2.961 (6.800)	-0.622	0.536
	Time Coverage (%)		0.288 (3.280)	1.385 (2.941)	-1.354	0.181
	Transition Probabilities (*10^3)	To A	2.764 (8.997)	-0.169 (13.525)	0.977	0.333
		To C	2.001 (15.860)	2.290 (14.635)	-0.073	0.942
		To D	-5.974 (18.267)	4.737 (28.254)	-1.723	0.090
Post−Pre Differences	Occurrence Rate (Hz)		0.010 (0.263)	-0.063 (0.421)	0.793	0.431
	**Mean Duration (ms)**		**2.145 (6.508)**	**-1.105 (4.895)**	**2.173**	**0.034**
	Time Coverage (%)		0.908 (3.482)	-0.698 (3.185)	1.850	0.070

for Microstate C	Transition Probabilities (*10³)	To A	4.807 (15.560)	-0.188 (13.536)	1.317	0.193
		To B	3.123 (11.877)	3.260 (15.058)	-0.039	0.969
		To D	-1.928 (10.912)	-3.762 (10.279)	0.665	0.509
Post−Pre Differences for Microstate D	Occurrence Rate (Hz)		-0.150 (0.429)	-0.101 (0.318)	-0.496	0.622
	Mean Duration (ms)		-1.034 (6.350)	1.726 (10.627)	-1.206	0.233
	Time Coverage (%)		-1.492 (3.421)	0.050 (4.999)	-1.378	0.174
	Transition Probabilities (*10³)	To A	-4.783 (12.519)	-3.294 (12.446)	-0.458	0.649
		To B	-3.143 (16.850)	3.675 (27.965)	-1.129	0.263
		To C	0.295 (10.740)	-3.011 (7.559)	1.371	0.176
